# Annotations

**Published:** 1909-09-18

**Authors:** 


### Annotations.

### Medical Fees at Inquests.

Among the many small points which may well engage the attention of the Commission, now sitting with regard to amendments in the Coroner's Law, some revision of the scale and method of distribution of fees to medical witnesses at inquests is of some importance to members of our profession. There is certainly room for improvement, for the present system is anomalous in several respects and frequently inflicts injustice. At the Wood Green Coroner's Court last week an old grievance arising out of the present state of the law was ventilated by a medical witness, and the Coroner expressed himself in sympathy with the medical man's contention. After attending the patient, upon whose body the inquest was held, for a fortnight, in his capacity as an honorary medical officer to the Wood Green Hospital, the medical practitioner was called upon to give evidence in the Coroner's Court without payment. Thus the medical witness in this instance, although in private practice, was forced by the law into the same position as that of a house officer who is called to give evidence at the inquest upon a patient who has died in a hospital with a resident staff. The medical practitioner said with some justice to the Coroner that, while his hospital services were a labour of love, his attendance at inquests was quite another matter. Resident medical officers have so long accepted the inevitable, that gratuitous evidence at all inquests, except upon persons brought in dead to hospital, scarcely now provokes even private comment; and it is very seldom that general practitioners attending local hospitals, or the consulting officers of larger institutions, make public complaint when compelled to attend without remuneration the inquests upon their hospital patients. But although silent resentment at petty injustice, and apathy over minor professional grievances, are common objects of our calling, and are perhaps in keeping with the dignity of the profession, it is still just as well, especially when a Commission is sitting upon the whole matter of the law relating to Coroner's inquests, that a voice should be raised to protest against anomalies of this sort which would easily admit of reform. No doubt the present method of remuneration was founded on principles of economy and upon some measure at least of common sense; but modifications could well be made which would secure equity without waste of public money, and the Wood Green Coroner hinted that the present Commission would probably see that this is done.

### Pure Consultants.

Honour and profit, says Aristotle, are as a rule incompatibles: whoever enjoys the first must not hope for the second, and *vice versâ*. That there is more than a gleam of truth in this in reference to the practice of medicine is a fact which has come home lately to a rather large number holding honorary hospital appointments in London. The present (it is to be hoped, only temporary) waning in prosperity of the metropolitan medical schools, which was recently part of our text, together with the ever-growing encroachments on the old, wide province of internal medicine made by surgeons, bacteriologists, specialists of all kinds, radiographers, and radio-therapists, have caused lucrative medical consultative practices to be few and far between. Quite a long time ago now it was said that whereas the physician used to arrive at the hospital door in a carriage and pair, while the surgeon trudged up on foot, their positions have become reversed. The passage of time has certainly not falsified this humorous putting of the case; and perhaps the future has in store the immunisator in a flying machine fitted up with a miniature laboratory. Then, too, in one or two quarters attempt, perhaps not highly successful, has been made to introduce some form of the curious practice in regard to fees known as dichotomy. It is, however, probably only coincidence that just lately, when emoluments have declined, a disposition should have been shown to stickle for the full honours attaching to the consultant status. Of course this can only affect London, because in the provinces there can scarcely be more than a few consultants undefiled by general practice, although probably Liverpool and Manchester and Birmingham and the rest are no great sufferers thereby. However, the proposal to mark the distinction more strongly seems, on the Aristotelian principle already quoted, to be equitable enough. There is danger, maybe, of casting by implication a stigma on the non-consultant. An opinion shows itself now and again that, as regards research, no good thing can come out of the Galilee of general practitioners. This does not matter so much (since, if truth is present, it must prevail) as what often goes along with it, namely, an easy tolerance of bad things of more fashionable origin. The lesson that the writer of *Confessio Medici* conveyed by the certainly adequate metaphor of Dædalus and Icarus was not unnecessary; and if practical failures in the higher branch of medical practice are to be regarded as oracles it will not be a good thing at all. After all, what the community wants of its doctors, as it wants of all classes of its members, is service of the general practitioner, plenty of sympathy with the sick and all-round ability in treating the common run of diseases; of the consultant, that he should be good at difficult cases—his strong point, like that of Mr. Shaw's Q.C., must be being right when other folk are wrong. And what it looks for from the research worker is results—news, a real advance, something novel; not academically correct proof that such-and-such a guinea-pig gave a positive reaction. Ability to furnish results, whether clinical or investigatory, should be recognised wherever it exists: hack-work is hack-work whoever did it, and however well it is done.

